# Hydrogen Metabolism in *Helicobacter pylori* Plays a Role in Gastric Carcinogenesis through Facilitating CagA Translocation

**DOI:** 10.1128/mBio.01022-16

**Published:** 2016-08-16

**Authors:** Ge Wang, Judith Romero-Gallo, Stéphane L. Benoit, M. Blanca Piazuelo, Ricardo L. Dominguez, Douglas R. Morgan, Richard M. Peek, Robert J. Maier

**Affiliations:** a Department of Microbiology, University of Georgia, Athens, Georgia, USA; b Division of Gastroenterology, Department of Medicine, Hepatology and Nutrition, Vanderbilt University School of Medicine, Nashville, Tennessee, USA; c Hospital de Occidente, Ministry of Health, Santa Rosa de Copan, Honduras; d Departments of Cancer Biology, Vanderbilt University School of Medicine, Nashville, Tennessee, USA

## Abstract

A known virulence factor of *Helicobacter pylori* that augments gastric cancer risk is the CagA cytotoxin. A carcinogenic derivative strain, 7.13, that has a greater ability to translocate CagA exhibits much higher hydrogenase activity than its parent noncarcinogenic strain, B128. A Δ*hyd* mutant strain with deletion of hydrogenase genes was ineffective in CagA translocation into human gastric epithelial AGS cells, while no significant attenuation of cell adhesion was observed. The quinone reductase inhibitor 2-*n*-heptyl-4-hydroxyquinoline-*N*-oxide (HQNO) was used to specifically inhibit the H_2_-utilizing respiratory chain of outer membrane-permeabilized bacterial cells; that level of inhibitor also greatly attenuated CagA translocation into AGS cells, indicating the H_2_-generated transmembrane potential is a contributor to toxin translocation. The Δ*hyd* strain showed a decreased frequency of DNA transformation, suggesting that *H. pylori* hydrogenase is also involved in energizing the DNA uptake apparatus. In a gerbil model of infection, the ability of the Δ*hyd* strain to induce inflammation was significantly attenuated (at 12 weeks postinoculation), while all of the gerbils infected with the parent strain (7.13) exhibited a high level of inflammation. Gastric cancer developed in 50% of gerbils infected with the wild-type strain 7.13 but in none of the animals infected with the Δ*hyd* strain. By examining the hydrogenase activities from well-defined clinical *H. pylori* isolates, we observed that strains isolated from cancer patients (*n* = 6) have a significantly higher hydrogenase (H_2_/O_2_) activity than the strains isolated from gastritis patients (*n* = 6), further supporting an association between *H. pylori* hydrogenase activity and gastric carcinogenesis in humans.

## IMPORTANCE

Hydrogen-utilizing hydrogenases are known to be important for some respiratory pathogens to colonize hosts. Here a gastric cancer connection is made via a pathogen’s (*H. pylori*) use of molecular hydrogen, a host microbiome-produced gas. Delivery of the known carcinogenic factor CagA into host cells is augmented by the H_2_-utilizing respiratory chain of the bacterium. The role of hydrogenase in carcinogenesis is demonstrated in an animal model, whereby inflammation markers and cancer development were attenuated in the hydrogenase-null strain. Hydrogenase activity comparisons of clinical strains of the pathogen also support a connection between hydrogen metabolism and gastric cancer risk. While molecular hydrogen use is acknowledged to be an alternative high-energy substrate for some pathogens, this work extends the roles of H_2_ oxidation to include transport of a carcinogenic toxin. The work provides a new avenue for exploratory treatment of some cancers via microflora alterations.

## INTRODUCTION

*Helicobacter pylori* is a pathogen that solely colonizes the mucosal surfaces of the human stomach (1). The persistent nature of the bacterium, combined with the highly inflammatory response of the host, is a key factor associated with the most severe manifestations of disease (2). There is very strong evidence that *H. pylori* infection increases the risk of gastric cancer (3, 4). *H. pylori* virulence factors play a role in determining the patterns of disease, with genetic differences affecting the clinical outcome of infection (5). One known *H. pylori* virulence factor that augments cancer risk is the *cag* pathogenicity island (PAI), which encodes a type IV secretion system (T4SS) and a CagA effector protein (6, 7). The *cag* T4SS injects CagA into host cells, where CagA is tyrosine phosphorylated and subsequently interferes with cell signaling pathway changes (8, 9). Infection with *cagA*-positive *H. pylori* strains is associated with an increased risk of developing gastric cancer (10–12). This has been confirmed by animal model experiments with Mongolian gerbils (13, 14). Thus, CagA has been designated a bacterial oncoprotein (7). However, many people colonized with *cagA*-positive *H. pylori* strains do not develop cancer (11), suggesting that other *H. pylori* constituents also affect disease risk.

In studying *H. pylori*-induced cancer in a Mongolian gerbil model, a carcinogenic *H. pylori* strain, 7.13, was selected from *in vivo* adaptation of noncarcinogenic strain B128 (15). Strain B128 is *cagA* positive, but it does not cause cancer in the gerbil model, unlike its derivative strain, 7.13 (15). Both strains B128 and 7.13 expressed similar levels of CagA when grown in broth alone, but the amount of CagA translocated into host cells by strain 7.13 was substantially greater than that for strain B128 (15). Further study indicated that inactivation of CagA in strain 7.13 attenuates the severity of *H. pylori*-induced inflammation and that development of gastric cancer is dependent on CagA (14).

*H. pylori* produce a hydrogen-utilizing hydrogenase, which provides the bacterium with a compact and high-energy noncarbon substrate for respiration-based energy generation (16, 17). Due to fermentative metabolism of normal colonic microflora, hydrogen gas is detected in animal tissues at supersaturated levels (5 logs increased over atmospheric levels) (17). Hydrogenase activity in *H. pylori*, while detected under all growth conditions, is dramatically increased when cells are provided with molecular hydrogen. A hydrogenase mutant strain of *H. pylori* is much less efficient in establishing colonization in mice (at 3 weeks postinoculation) (17).

In the present study, we found that the carcinogenic strain 7.13 has a much higher level of hydrogenase activity than parent strain B128, suggesting a potential link between hydrogen metabolism and carcinogenesis. The 7.13 Δ*hyd* hydrogenase deletion mutant strain has almost lost the ability to translocate CagA into host cells, suggesting that *H. pylori* hydrogen metabolism may induce gastric cancer via promotion of CagA translocation. In a gerbil model of infection, we observed that the Δ*hyd* strain produces a significantly lower level of inflammation than wild-type (WT) strain 7.13, further supporting the notion that hydrogen metabolism plays an important role in the etiology of *Helicobacter*-mediated gastric cancer. Furthermore, we provided evidence showing that the clinical strains isolated from cancer patients have an overall higher hydrogenase activity than the strains isolated from gastritis-only patients.

## RESULTS

### The carcinogenic *H. pylori* strain 7.13 has a high level of hydrogenase activity.

To search for potential virulence factors in strain 7.13 that contributed to its carcinogenic ability, we determined the hydrogenase activity of strain 7.13 compared to that of the parental strain B128 as well as to other well-defined *H. pylori* strains. The strains were grown either without or with H_2_ (10%) added to the closed gas culture system (Table 1). As expected, all strains expressed a significantly higher level of hydrogenase activity (H_2_ uptake or oxidation) when grown under the condition with H_2_ added than without H_2_ added to the atmosphere. Strikingly, strain 7.13 showed a much higher level of hydrogenase activity than other strains (3-fold higher than its parent strain, B128). Actually, this is the highest level of hydrogenase activity among all the *H. pylori* strains we have analyzed. As expected, the Δ*hyd* strains in which the entire *hydABCDE* operon was deleted had no detectable hydrogenase activity.

**TABLE 1:** Hydrogenase activity of *H. pylori* strains in this study

*H. pylori* strain	Hydrogenase activity (nmol/min/10^9^ cells):	
	Without added H_2_	With H_2_ (10%) added
X47	8.0 ± 1.5	21.2 ± 1.8
B128	12.2 ± 0.8	16.3 ± 2.5
7.13	43.5 ± 6.6	69.1 ± 4.9
7.13 Δ*hyd*	<0.1	<0.1
26695	15.5 ± 2.2	38.3 ± 3.4
26695 Δ*hyd*	<0.1	<0.1

### *H. pylori* hydrogenase affects translocation of CagA into host cells.

**FIG 1:**
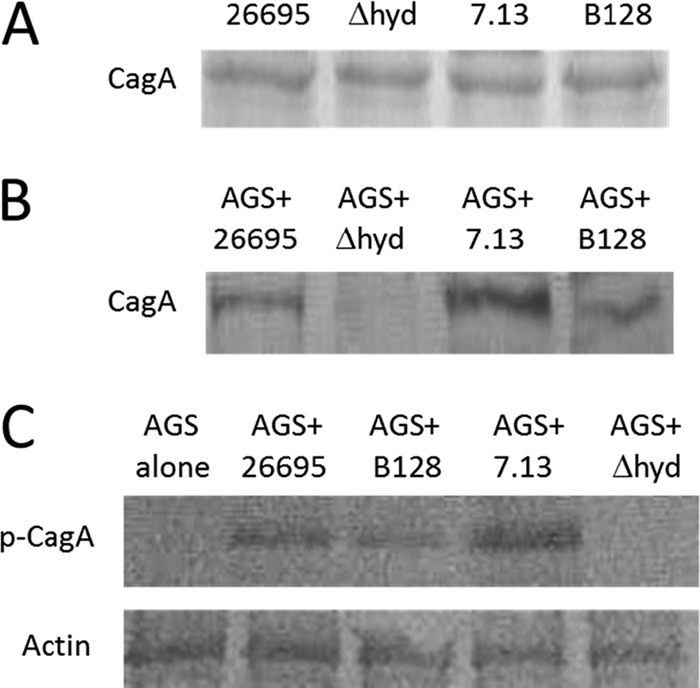
*H. pylori* hydrogenase enhances CagA translocation into AGS human gastric cells. Western blot analysis using anti-CagA antibody for different *H. pylori* strains grown in broth alone (A). Lysates of AGS cells after coculture with different *H. pylori* strains were used for Western blot analysis using anti-CagA antibody (B) or anti-phosphotyrosine (α-PY99) antibody (C).

The ample hydrogenase activity observed for the carcinogenic strain 7.13 prompted us to propose that H_2_ metabolism may be one factor connected to carcinogenesis. Particularly, H_2_ use by *H. pylori* is hypothesized to augment energy-requiring cancer etiology-related processes; one of these may be CagA translocation. To test this hypothesis, we assayed CagA translocation for strains 26695 (reference strain), B128, and 7.13, as well as the 7.13 Δ*hyd* mutant strain (Fig. 1). First, we examined CagA expression in these strains when grown in broth alone (Fig. 1A). Similar levels of CagA expression were observed among strains, indicating that deletion (or change) of hydrogenase activity did not affect CagA expression. Next, *H. pylori* and AGS human gastric cells were cocultured under the H_2_-added (10% partial pressure) condition. CagA translocation was assessed by Western blotting using either anti-CagA (Fig. 1B) or anti-PY99 antibody (Fig. 1C); both experiments produced similar results. Strain 7.13 translocated a significantly larger amount of CagA into AGS cells than B128, while strain 26695 translocated an intermediate amount of CagA. The Δ*hyd* mutant strain has almost lost the ability to translocate CagA into AGS cells, indicating that hydrogenase activity plays an important role in CagA translocation into host cells. When H_2_ was omitted from the translocation experiment, a similar result was observed, although the amounts of CagA translocated by WT cells were significantly lower than those under the H_2_-added condition (data not shown).

Translocation of CagA is dependent on *H. pylori* adherence to host cells (8). Carcinogenic strain 7.13 bound significantly more avidly (>1.5 log-fold increase) to epithelial cells than did strain B128, suggesting that one mechanism through which *H. pylori* strain 7.13 can deliver more CagA into epithelial cells may be through its ability to adhere more avidly to host cells (15). We examined whether hydrogenase affects *H. pylori* adherence to host cells. The bacterial adherence assay with AGS cells revealed no significant difference between the binding abilities of the Δ*hyd* mutant strain and its parent strain, 7.13 (data not shown).

### CagA translocation is mechanistically connected to hydrogen metabolism of *H. pylori*.

**FIG 2:**
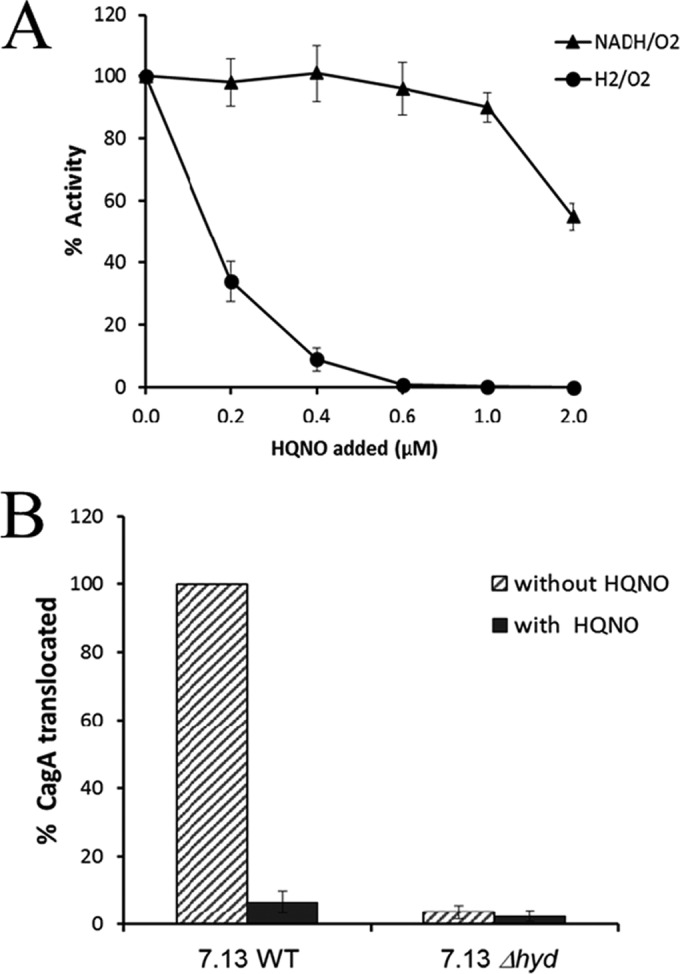
Both the hydrogenase activity and CagA translocation ability of *H. pylori* are specifically inhibited by HQNO. (A) Permeabilized fresh *H. pylori* 7.13 cell suspensions were used for determining the activities of hydrogenase (H_2_/O_2_) or NADH oxidase (NADH/O_2_) in the presence of different concentrations of HQNO. The activity is expressed as the percentage relative to that without HQNO, and the data are averages (with standard deviations) from three independent determinations. (B) Lysates of AGS cells after coculture with the permeabilized *H. pylori* 7.13 WT or Δ*hyd* mutant strain in the absence or presence of 0.5 µM HQNO were used for Western blot analysis using anti-CagA antibody. The data shown are averages (with standard deviations) from densitometric analysis of three Western blots and are expressed as percentages relative to that of the 7.13 WT strain without HQNO.

Upon connecting H_2_ oxidation to CagA translocation, it was of interest to know the mechanism powering this cytotoxin delivery event. It is known that CagA is translocated in a type IV secretion process that requires energy input (18, 19). CagA translocation could be energized by either hydrogenases that “split” molecular H_2_ or by NAD(P)H dehydrogenases that act as proton pumping enzymes (20). The quinone reductase site on bacterial hydrogenases is highly sensitive to the quinone (Q) analogue 2-*n*-heptyl-4-hydroxyquinoline-*N*-oxide (HQNO) (21, 22), so a low level of HQNO was used to specifically disrupt the respiratory chain from H_2_ in permeabilized cells. As shown in Fig. 2A, submicromolar levels of HQNO strongly inhibited H_2_ oxidation to O_2_ as the terminal acceptor, without affecting NADH oxidation.

Using this specific Q-reductase inhibitor (HQNO), we performed additional CagA translocation assays. *H. pylori* cells (7.13 WT or 7.13 Δ*hyd* mutant) were first treated for 10 min with the sublethal dose (3 mg/ml) of lactoferrin to permeabilize the outer membrane (23), thus facilitating the access of HQNO to hydrogenase at the cytoplasmic membrane and periplasm-facing components. The cells were then washed with PBS. The permeabilized *H. pylori* cells were then incubated (under a 10% H_2_ atmosphere condition) with AGS cells, in the absence or presence of 0.5 µM HQNO, to assess the level of CagA translocation. Treatment of *H. pylori* cells with lactoferrin plus HQNO had no significant effect on cell viability (data not shown). As the positive control, the WT strain 7.13 translocated a large amount of CagA into AGS cells in the absence of HQNO. With addition of 0.5 µM HQNO, more than 90% of the amount of CagA translocation in WT strain 7.13 was inhibited (Fig. 2B). As expected, the 7.13 Δ*hyd* mutant strain translocated a very small amount of CagA into AGS cells, either with or without the HQNO treatment. The results indicated the H_2_-oxidation-mediated transmembrane potential contributes to the CagA translocation process.

### *H. pylori* hydrogenase affects efficiency of DNA transformation.

In addition to the Cag T4SS, *H. pylori* has another T4SS encoded by the *comB* locus. The ComB T4SS is responsible for transport of DNA into *H. pylori* cells and thus is designated a DNA transformation competence apparatus (24–26). To test if *H. pylori* hydrogen metabolism also plays a role in facilitating ComB function, we compared the DNA transformation frequency of the 7.13 Δ*hyd* mutant strain to that of WT 7.13.

As described previously (27–29), we used two different types of DNA to examine DNA transformation of *H. pylori*. A specific A-to-G mutation in the *H. pylori* rpoB gene (*rpoB3* allele) confers rifampin resistance (30). A 330-bp PCR fragment containing this specific mutation at the center of the fragment was used to transform *H. pylori* strains by using rifampin resistance as a selective marker. Another type of DNA used for transformation was the sequence of the *H. pylori* acnB gene (a housekeeping gene [1.1 kb]) in which a kanamycin resistance cassette (Kan [1.4 kb]) was inserted at the center (*acnB*::Kan).

The results for transformation are shown in Table 2. Using *rpoB3* as the donor DNA, the 7.13 Δ*hyd* mutant had a transformation frequency of 3.89 × 10^−5^, which was approximately 8-fold lower than that for the 7.13 WT strain (3.27 × 10^−4^). Using *acnB*::Kan as the donor DNA, wild-type *H. pylori* strain 7.13 had a transformation frequency of 1.27 × 10^−5^. In contrast, the transformation frequency for the 7.13 Δ*hyd* mutant was 1.4 × 10^−6^, which is 9-fold lower than that of the WT strain. For both types of DNA donor (*rpoB3* and *acnB*::Kan), the mutant strain data (transformation frequency) are significantly lower than those of the WT strain, according to Student’s *t* test (*P* < 0.01). These results provided evidence that hydrogenase plays a significant role in the DNA transformation process of *H. pylori*.

**TABLE 2:** DNA transformation frequency

*H. pylori* strain	Transformation frequency with donor DNA^a^:	
	*rpoB3* (330 bp)	*acnB*::Kan (2.5 kb)
7.13 WT	32,680 ± 5,360	1,270 ± 196
7.13 Δ*hyd*	3,890 ± 620	140 ± 17

a The two *H. pylori* strains were incubated under a condition involving 10% hydrogen gas. The transformation frequencies are presented as the number of transformants (resistant colonies) per 10^8^ recipient cells. Data are means ± standard errors from three independent determinations.

### Hydrogen metabolism of *H. pylori* affects carcinogenesis in an animal model.

*H. pylori* persistence, combined with the highly inflammatory response of the host, is associated with the most severe manifestations of disease (2). For testing *H. pylori* infection and gastric cancer development, the Mongolian gerbil model is well established. *H. pylori* strain 7.13 was shown to have a much higher ability (than parental strain B128) to induce gastric cancer in this animal model, and the development of gastric dysplasia and adenocarcinoma is dependent on the bacterial virulence factor CagA (14, 15). In this study, we assessed the effects of H_2_ use on *H. pylori* colonization and inflammation in gerbils by comparing the 7.13 parent to its Δ*hyd* isogenic mutant strain.

**FIG 3:**
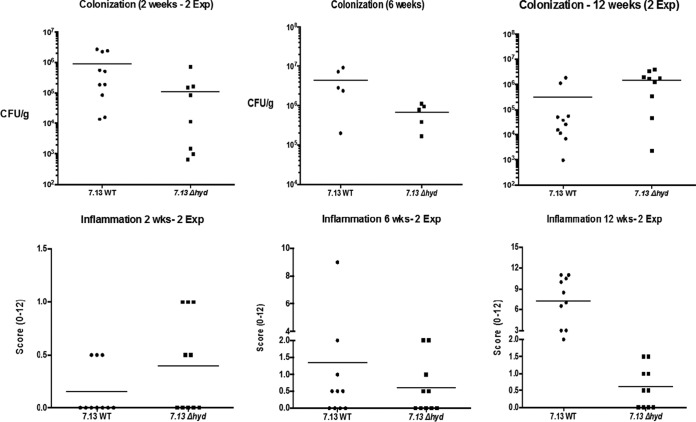
Severity of inflammation within the gastric antra of Mongolian gerbils inoculated with *H. pylori* is dependent on hydrogenase. Mongolian gerbils were inoculated with the *H. pylori* 7.13 WT or Δ*hyd* mutant strain, and the stomachs were examined 2, 6, and 12 weeks after inoculation. Colonization data (upper panel) are presented as a scatter plot of CFU per gram of stomach as determined by plate counts. Indices of inflammation (lower panel) are presented as scores (0 to 12) examined by a single pathologist blind to the treatment groups. The horizontal bars represent the geometric means for each group.

**FIG 4:**
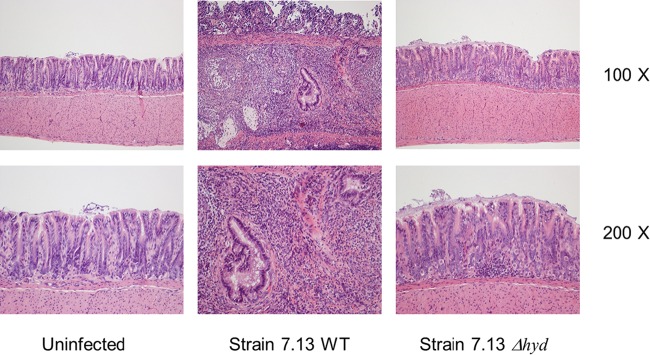
The development of gastric dysplasia and adenocarcinoma is dependent on *H. pylori* hydrogenase. Representative H&E-stained panels are shown for 12 weeks postinfection with the *H. pylori* 7.13 WT or Δ*hyd* mutant strain. Magnification: upper panel, ×100; lower panel, ×200. Invasive adenocarcinoma developed in some gerbils infected with the 7.13 WT strain. No dysplasia or adenocarcinoma developed in gerbils infected with the 7.13 Δ*hyd* mutant strain. No gastric injury was seen in uninfected control gerbils.

The experiment was performed 2, 6, and 12 weeks after *H. pylori* inoculation into the animals. Five gerbils were used for each group, and the experiment was repeated once (10 gerbils in total for each time point) (Fig. 3). At 2 and 6 weeks, the colonization efficiency of the Δ*hyd* strain was lower than that for the parent 7.13 strain (upper panel), which is in agreement with the previous observations in mice (17), suggesting that *H. pylori* hydrogenase facilitates the pathogen’s initial infection. However, at 12 weeks postinoculation, the Δ*hyd* strain colonized somewhat better than the WT strain, which could be a consequence of the lowered level of host inflammatory response (described below). At 2 and 6 weeks, very low levels of inflammation were observed in gerbils, and the difference between WT 7.13 and Δ*hyd* mutant strain infection was not statistically significant (Fig. 3, lower panel). Strikingly, all of the gerbils infected with WT 7.13 at 12 weeks exhibited a very high level of inflammation, while the inflammation scores for the Δ*hyd* strain-infected gerbils remained low, despite similar colonization densities, similar to those at 2 and 6 weeks postinoculation. At 12 weeks postinoculation, 2 out of 10 gerbils infected with WT 7.13 developed low-grade dysplasia, and 3 developed invasive adenocarcinoma. None of the gerbils infected with the Δ*hyd* strain developed dysplasia or adenocarcinoma (Fig. 4). These results indicated that *H. pylori* hydrogenase plays an important role in inducing inflammation and carcinogenesis in the host.

### *H. pylori* hydrogenase activity is associated with gastric carcinogenesis in humans.

To increase the significance of the results beyond the B128 and 7.13 model strains, we examined the hydrogenase activities as well as CagA status for other clinical *H. pylori* isolates from persons residing in Honduras, Central America, and in Japan. *H. pylori*-infected subjects were categorized into those with nonatrophic gastritis only (*n* = 6) or those with gastric adenocarcinoma (*n* = 6). The *in vitro* hydrogenase activities (when strains are grown under a 10% H_2_ atmosphere condition) of these 12 categorized *H. pylori* strains are shown in Table 3. Strikingly, the 6 cancer strains have an overall higher hydrogenase activity (mean, 48.7 nmol/min/10^9^ cells) than the 6 non-cancer strains (mean, 19.9 nmol/min/10^9^ cells). According to a Wilcoxon rank test, the difference between the two groups is statistically significant (*P* < 0.01). We found that all 6 cancer strains and 4 of the non-cancer strains are CagA positive. Two of the non-cancer strains (T135 and T151) are CagA negative, as determined by Western blotting with anti-CagA antibody, although they were *cagA* positive by PCR genotyping. Interestingly, the two CagA-negative strains (T135 and T151) have intermediate levels of hydrogenase activity, higher than those of CagA-positive non-cancer strains. Although this is a limited data set, these results further support the notion that hydrogen metabolism plays a role in *H. pylori*-induced gastric carcinogenesis through facilitating CagA translocation into host cells.

**TABLE 3:** Hydrogenase activity of categorized clinical strains of *H. pylori*

*H. pylori* strain	Patient condition	CagA status^a^	Hydrogenase activity (nmol H_2_ used/min/10^9^ cells)^b^
T21	Gastritis only	+	19.7 ± 2.9
T8	Gastritis only	+	11.5 ± 2.1
T9	Gastritis only	+	5.7 ± 2.0
T135	Gastritis only	−	25.1 ± 3.9
T151	Gastritis only	−	37.6 ± 3.9
T152	Gastritis only	+	19.6 ± 3.5
C18	Gastric cancer	+	54.5 ± 3.9
C11	Gastric cancer	+	44.7 ± 8.9
C108	Gastric cancer	+	25.4 ± 6.2
C10	Gastric cancer	+	63.9 ± 7.7
C112	Gastric cancer	+	60.4 ± 6.4
C116	Gastric cancer	+	43.1 ± 7.8

a CagA status was determined by Western blotting using anti-CagA antibody.

b Hydrogenase activity is expressed as nanomoles of H_2_ used per minute per 10^9^ cells. Data are means ± standard errors from three independent determinations.

## DISCUSSION

In studying *H. pylori*-induced cancer in a Mongolian gerbil model, a carcinogenic *H. pylori* strain, 7.13, was selected from *in vivo* adaptation of noncarcinogenic strain B128 (15). Subsequently, some potential factors that may confer the carcinogenic ability of strain 7.13 have been identified, which include an outer membrane adhesion protein (OipA) and a peptidoglycan deacetylase (PgdA), in addition to CagA (14, 31, 32). In this study, we provided evidence that the high level of hydrogenase activity in strain 7.13 also contributes to its carcinogenic ability. This notion is further supported by the findings that the clinical strains isolated from cancer patients have an overall higher hydrogenase activity than the strains isolated from gastritis-only patients (Table 3). It seems that multiple factors related to carcinogenic ability are increased in strain 7.13.

All *H. pylori* strains contain a hydrogen-utilizing hydrogenase, which provides the bacterium with a compact and high-energy substrate for respiratory-based energy generation (16, 17, 33). On binding and then “splitting” of hydrogen gas by membrane-associated nickel-containing hydrogenases, the energy contained in the low-potential electrons is conserved by a combination of transmembrane potential and proton gradient coupling mechanisms (34). The H_2_ concentration in gastric mucosa of live mice was shown to be 10 to 50 times higher than the *H. pylori* affinity for H_2_ binding by its hydrogenase (17, 35). While we do not know the H_2_ concentrations achieved in the gastric mucus of humans, hydrogen gas in the human gut is known to be produced by primary fermenters of the microbiota (36, 37). Molecular hydrogen is thus far the only known intestinal microbiota-produced growth substrate that is known to be present in the mucus and epithelial tissue of the stomach. Results presented in the present study provide a connection between gut commensal microbiota and *Helicobacter*-mediated carcinogenesis via a single gaseous metabolite.

While there is a clear difference in hydrogenase activity between strain 7.13 and its parental strain, B128, the actual underlying cause is still under investigation. In *H. pylori*, hydrogenase structural genes are contained in the *hydABCDE* operon; *hydA* encodes the [Fe-S]-containing small subunit, *hydB* encodes the [Ni-Fe] large subunit, and *hydC* encodes the cytochrome *b* subunit (38); while two other components, HydD and HydE, are involved in localizing the hydrogenase complex to the membrane (39). In addition, *H. pylori* strains possess many accessory proteins (HypA, -B, -C, -D, -E, and -F and SlyD) that are required for maturation of the apoenzyme (39–44). Other factors related to efficient hydrogenase maturation include a nickel-specific permease (NixA) (45), a histidine-rich heat shock protein, HspA (GroES) (46), two other histidine-rich, nickel-binding proteins (Hpn and Hpn-like) (47, 48), and the twin-arginine translocation (Tat) system (49), as well as factors involved in the Fe-S cluster relay and in iron transport (50). Furthermore, the expressions of the hydrogenase-related proteins were shown to be regulated (directly or indirectly) at different levels by several regulatory proteins, including the nickel-dependent regulator (NikR) (51, 52), the ferric uptake regulator (Fur) (53, 54), the two-component signal transduction system ArsRS (55), and the posttranscriptional regulator aconitase (56). Any of these factors could contribute to the expression or augmentation of hydrogenase activity.

To identify proteins that may confer the carcinogenic ability of strain 7.13, a proteome analysis was conducted (31), but no hydrogenase-related proteins were identified in the list of differently expressed proteins between strains 7.13 and B128. H_2_-utilizing hydrogenases have high catalytic efficiency and are typically made in very small protein amounts; consequently two-dimensional (2D) gel analysis may not reveal any significant differences in hydrogenase protein levels between strain B128 and strain 7.13. Furthermore, Western blot analysis revealed no significant difference for the expression level of HydA, HypA, or HypB between strains B128 and 7.13 (data not shown). The whole-genome sequences of strains B128 and 7.13 are available for direct comparison, although the genome sequence of 7.13 is incomplete (57). The majority of the genes related to hydrogenase expression/activity mentioned above (e.g., *hydA*, -*B*, -*C*, and -*D*, *hypA*, -*B*, -*C*, and -*F*, and *slyD*, *nixA*, *hspA*, *fur*, and *acnB*) are identical in both strains, whereas a few other genes (e.g., *nikR*, *hpn*, and *hpn-*like) could not be identified from the 7.13 genome sequence due to lack of complete sequence information. Of interest, sequence variations (mutations) between the two strains were observed in *tatC*, *hypD*, and *hypE*, resulting in alteration of the encoded proteins. TatC is an integral component of the Tat system in *H. pylori*, and it plays an essential role in translocating hydrogenase subunit HydA into the cytoplasmic membrane (49). While HypA and HypB are required for maturation/activation of both nickel-containing enzymes urease and hydrogenase, HypD and HypE are specifically required for hydrogenase (39–42, 58). The altered TatC, HypD, and HypE proteins in strain 7.13 (compared to B128) may be more efficient in translocation/maturation of hydrogenase, perhaps resulting in high hydrogenase activity. Studies are under way to further explore the genetic bases for differences in hydrogenase activities among *H. pylori* strains.

The well-characterized *H. pylori* virulence factor that enhances cancer risk is the *cag* PAI, which that encodes a T4SS and CagA effector protein. Our data demonstrated that the 7.13 Δ*hyd* strain has little ability to translocate CagA into host cells in the presence of molecular hydrogen (Fig. 1), suggesting that *H. pylori* hydrogen metabolism may provide energy for the Cag T4SS to promote CagA translocation. Hydrogenases that “split” molecular H_2_ operate as unique redox “half-loop” enzymes (20), and quinone (Q) reduction after H_2_ splitting plays a key role in the proton translocation mechanism. By separating the proton motive force generated by hydrogenase through Q reductase versus that from electrons supplied by NAD(P)H, we assessed the contribution of the H_2_-specific transmembrane potential to CagA translocation. The results in Fig. 2 support the hypothesis that H_2_-mediated CagA translocation can be linked to the half-loop Q mechanism associated with H_2_ splitting. *H. pylori* produces abundant CagA protein; however, only a relatively small amount of CagA is translocated into host cells under *in vitro* infection conditions (apparently under conditions without hydrogen added) (59). Our recent proteomic study showed that the hydrogenase large subunit HydB was up-expressed in the presence of H_2_ (60). As ample amounts of hydrogen gas are likely often available within the human gastric-intestinal tract (61–63), *Helicobacter* hydrogenase expression would be H_2_ augmented, and the amount of CagA translocation mediated by hydrogenase could be relatively high *in vivo*.

T4SSs are macromolecular devices that bacteria use to transport various macromolecules across the cell envelope; this would include protein, DNA, or nucleic acid-protein complexes (64). Different groups of T4SS have been identified in *H. pylori*. The most conserved T4SS in *H. pylori* is the ComB system that mediates the import and integration of environmental DNA fragments into its genome (24). The *cag* T4SS, present in the virulent *H. pylori* strains, is specialized for the transfer of bacterial components, such as CagA protein, peptidoglycan, and DNA, into host epithelial cells (8, 65, 66). Gram-negative T4SSs employ one or more ATPases (VirB4 and VirB11 homologs) to energize early steps of biogenesis or substrate transfer (64). The Cag T4SS powering machinery is composed of three cytoplasmic ATPases, CagE (VirB4), Cagα (VirB11), and Cagβ (VirD4), which supply the energy necessary to assemble the apparatus and secrete CagA (18). While the present study indicates that the *H. pylori* Δhyd strain has a greatly decreased ability to translocate CagA, we do not know whether assembly of the Cag T4SS is affected in the Δ*hyd* strain. The ComB T4SS has only one ATPase, ComB4 (VirB4) (25). Our data on DNA transformation (Table 2) suggest that *H. pylori* hydrogen metabolism may also provide energy for the ComB T4SS to facilitate DNA uptake. Currently, it is unknown whether DNA transformation into *H. pylori* is dependent on the transmembrane potential. Further studies are needed to investigate the effects of hydrogenase on transport of macromolecules such as peptidoglycan and DNA. In addition to providing energy for the T4SS, *H. pylori* hydrogenase may have other important physiological roles. For example, recent data from our laboratory revealed a new aspect of the metabolic flexibility in *H. pylori*, whereby molecular hydrogen use (energy) is coupled to carbon dioxide fixation (carbon acquisition) via a described carbon fixation enzyme (60).

A hallmark of acute and chronic *H. pylori* infection is intense inflammation in the gastric mucosa, which is strongly enhanced by the activity of Cag T4SS. The *cag* PAI-positive strains are implicated in the increased release of various inflammatory cytokines from the host cells, including interleukin-8 (IL-8) (67), whereas an isogenic mutant with a defect in T4SS is unable to elicit this response (68). Our *in vivo* gerbil model data (Fig. 3) demonstrated that the Δ*hyd* strain produced a significantly lower level of inflammation and no neoplastic transformation compared to its parent strain, 7.13. This is at least in part related to the observation that the Δ*hyd* strain has a greatly decreased ability to translocate CagA into host cells. Collectively, these results suggest that *H. pylori* hydrogen metabolism may represent a new factor that enhances gastric cancer risk. In addition to generating new ideas about how *H. pylori* causes diseases, this study provides potential options for new screening and treatment of *H. pylori*-caused diseases. For example, the risk of developing gastric cancer could be predicted by prospectively testing *H. pylori* strains’ *cagA* status in combination with the hydrogenase activity. Further studies are warranted to investigate how environmental factors (such as diet) could impact the hydrogen production/utilization of microbiota.

## MATERIALS AND METHODS

### *H. pylori* culture conditions.

*H. pylori* cells were cultured on brucella agar (Difco) plates supplemented with 10% defibrinated sheep blood (BA plates). Chloramphenicol (50 µg/ml) was added to the medium for culturing mutants. Cultures of *H. pylori* were grown microaerobically at 37°C in an incubator under continuously controlled levels of oxygen (4% partial pressure O_2_ and 5% CO_2_, with the balance N_2_).

### Construction of the *H. pylori* 7.13 Δ*hyd* mutant.

Construction of *H. pylori* Δhyd mutants in strains 26695 and 43504 was previously described (49). Briefly, overlapping PCR and allele-exchange mutagenesis were used to generate the Δ*hyd* deletion mutant, in which the entire *hyd* operon (*hydABCDE* [genes *hp0631* to *hp0635* in strain 26695]) was deleted and replaced by a chloramphenicol acetyltransferase gene (*cat*) cassette. In this study, the DNA construct was used to transform strain 7.13 to obtain the 7.13 Δ*hyd* mutant.

### Enzyme assays.

Cells were grown in the presence of H_2_ (in sealed anaerobic jars with CampyPak Plus microaerophilic envelopes; Becton, Dickinson, Sparks, MD), harvested and resuspended in phosphate-buffered saline (PBS), cell density (optical density at 600 nm [OD_600_]) was measured, and hydrogen uptake was monitored using a previously described amperometric method (16, 17). Hydrogenase activity was expressed as nanomoles of H_2_ used per minute per 10^9^ cells. For the HQNO inhibition experiments, oxygen was present in the sealed amperometric chamber at levels of 40 to 75 µM and cells were first permeabilized for 10 min with lactoferrin (23). NADH was added to 1 mM, and the O_2_ uptake rate was determined (69). Results represent 2 to 4 independently grown cultures, each with at least 3 assay replicates.

### CagA translocation assay.

AGS human gastric cells (from ATCC) were grown in Dulbecco’s modified Eagle’s medium (DMEM [from ATCC]) with 10% fetal bovine serum for 24 h to subconfluency. *H. pylori* was added at a multiplicity of infection of 100:1, and cells were further incubated for 8 h at 37°C in the presence of 5% CO_2_. The cell cocultures were washed with PBS until no *H. pylori* adhered on cells under the microscope. The scraped cells were lysed in NP-40 lysis buffer containing Complete Mini EDTA-free protease inhibitor (Roche). Proteins in the lysates were separated using sodium dodecyl sulfate-polyacrylamide gel electrophoresis (SDS-PAGE) and then transferred onto a nitrocellulose membrane for Western blotting. CagA translocated into AGS cells were detected by using anti-CagA antibody (Santa Cruz). Alternatively, levels of phosphorylated CagA were determined by using an anti-phosphotyrosine antibody (α-PY99 [Santa Cruz]), and actin levels as controls were determined using an antiactin (C-11) antibody (Santa Cruz). Primary antibodies were detected using goat anti-rabbit (Santa Cruz) horseradish peroxidase-conjugated secondary antibodies and visualized by a chemiluminescence detection system.

### Bacterial adherence assay.

AGS cells were grown for 24 h to subconfluency, and *H. pylori* cells were added to cells at a bacterium/cell ratio of 100:1. After 4 h, *H. pylori*-gastric cell cocultures were washed with PBS three times to remove nonadherent bacteria. The AGS cells were lysed with ice-cold PBS plus 0.1% saponin for 5 min. Serial 10-fold dilutions of cell lysates were cultured on BA plates for 3 to 5 days at 37°C under microaerobic conditions to count the number of *H. pylori* colonies.

### Determination of DNA transformation frequency.

*H. pylori* strains were grown on BA plates to late log phase, and the cells were suspended in PBS at a concentration of approximately 10^8^ per ml (recipient cells for transformation). A 30-µl cell suspension sample was mixed with 100 ng of donor DNA and spotted onto a BA plate. After incubation for 18 h under the microaerobic condition at 37°C, the transformation mixture was harvested and suspended in 1 ml PBS. One hundred-microliter portions of the suspension (or appropriate dilution as needed) were plated onto either BA plates or BA plates containing selective antibiotic (20 µg/ml rifampin or 40 µg/ml kanamycin, depending on the donor DNA used). The plates were incubated for 4 days under the microaerobic condition at 37°C, and the numbers of colonies were counted. The transformation frequency was determined by the number of resistant colonies divided by the total number of CFU. In a normalized DNA transformation assay, the frequency of transformation is expressed as the number of transformants per 10^8^ recipient cells. As negative controls, *H. pylori* strains with no DNA added were tested under this assay condition; no antibiotic-resistant colonies were observed.

### Animals and *H. pylori* challenge.

The Institutional Animal Care Committee of Vanderbilt University approved all procedures. Multiple cohorts of male Mongolian gerbils, ages 5 to 7 weeks, were orogastrically challenged with *H. pylori* wild-type strain 7.13 or isogenic mutant strain 7.13 Δ*hyd* (10^9^ CFU/gerbil) and were euthanized 2, 6, or 12 weeks postinoculation. One-half of the stomach, as linear strips extending from the squamocolumnar junction through proximal duodenum, was fixed in 10% neutral buffered formalin, paraffin-embedded, and stained with hematoxylin and eosin (H&E), and indices of inflammation and injury were scored from 0 to 12 by a single pathologist blind to the treatment groups. The other half was divided into two strips, and one was homogenized in sterile PBS, serially diluted, plated on selective Trypticase soy agar–5% sheep blood plates containing vancomycin (20 µg/ml), bacitracin (200 µg/ml) (Calbiochem, San Diego, CA), nalidixic acid (10 µg/ml), and amphotericin B (2 µg/ml) (Sigma Chemical Co., St Louis, MO), and incubated for 5 to 7 days for examination of *H. pylori* colonization. One-fourth of the stomach was stored at −80°C for further studies (DNA and RNA extraction).

### *H. pylori* strains from humans.

Six strains were harvested from Japanese patients, who had gastric cancer or gastritis only as described previously (70). Six *H. pylori* strains were selected at random from an ongoing population-based, case-control gastric cancer epidemiology initiative in western Honduras. Honduras has among the highest gastric cancer incidence rates in Latin America and the western hemisphere (71, 72). *H. pylori* infection is endemic in the population, at over 82 to 85% (73, 74).
